# Re-classification of *Clavibacter michiganensis* subspecies on the basis of whole-genome and multi-locus sequence analyses

**DOI:** 10.1099/ijsem.0.002492

**Published:** 2017-11-21

**Authors:** Xiang Li, James Tambong, Kat (Xiaoli) Yuan, Wen Chen, Huimin Xu, C. André Lévesque, Solke H. De Boer

**Affiliations:** ^1^​ Canadian Food Inspection Agency (CFIA), Charlottetown Laboratory, 93 Mount Edward Road, Charlottetown, PE C1A 5T1, Canada; ^2^​ Agricultural and Agri-Food Canada (AAFC), Ottawa Laboratory, 960 Carling Ave, Ottawa K1A 0C6, Canada

**Keywords:** *Clavibacter capsici*, *Clavibacter michiganensis*, *Clavibacter insidiosus*, *Clavibacter nebraskensis*, *Clavibacter sepedonicus*, *Clavibacter tessellarius*

## Abstract

Although the genus *Clavibacter* was originally proposed to accommodate all phytopathogenic coryneform bacteria containing B2γ diaminobutyrate in the peptidoglycan, reclassification of all but one species into other genera has resulted in the current monospecific status of the genus. The single species in the genus, *Clavibacter michiganensis*, has multiple subspecies, which are all highly host-specific plant pathogens. Whole genome analysis based on average nucleotide identity and digital DNA–DNA hybridization as well as multi-locus sequence analysis (MLSA) of seven housekeeping genes support raising each of the *C. michiganensis* subspecies to species status. On the basis of whole genome and MLSA data, we propose the establishment of two new species and three new combinations: *Clavibacter capsici* sp. nov., comb. nov. and *Clavibacter tessellarius* sp. nov., comb. nov., and *Clavibacter insidiosus* comb. nov., *Clavibacter nebraskensis* comb. nov. and *Clavibacter sepedonicus* comb. nov.

The genus *Clavibacter* was originally proposed by Davis *et al.* [1] to accommodate all phytopathogenic coryneform bacteria containing B2γ diaminobutyrate in the peptidoglycan. This genus originally included six plant pathogenic species: *Clavibacter michiganensis*, *Clavibacter iranicum, Clavibacter rathayi, Clavibacter toxicus, Clavibacter tritici* and *Clavibacter xyli*. Subsequently, the grass-specific pathogens, *C. iranicum, C. rathayi, C. toxicus* and *C. tritici*, were reclassified into the genus *Rathayibacter* on the basis of DNA–DNA hybridization and their unique menaquinone structures [2]. The two subspecies of *C. xyli* were placed in the genus, *Leifsonia* [3, 4]. Currently, the genus *Clavibacter* consists of only one species, *C. michiganensis,* which is subdivided into seven subspecies of plant pathogenic bacteria with narrow host specificities and two subspecies with close association with tomato and pepper seeds. Five of the subspecies comprise well-known pathogens, namely, *C. michiganensis*subsp.*michiganensis* (Cmm; bacterial canker and wilt of tomato), *C. michiganensis*subsp.*sepedonicus* (Cms; bacterial ring rot of potato), *C. michiganensis*subsp.*insidiosus* (Cmi; wilting and stunting in alfalfa), *C. michiganensis*subsp.*nebraskensis* (Cmn; wilt and blight of maize), and *C. michiganensis*subsp.*tessellarius* (Cmt; leaf freckles and leaf spots in wheat). More importantly, the first three subspecies are quarantine or regulated pathogens of important agricultural crops in many countries. Recently, *C. michiganensis*subsp.*phaseoli* was described as the causal agent of bacterial leaf yellowing on bean [5] and *C. michiganensis*subsp.*capsici* (Cmc) as the causal agent of bacterial canker on pepper [6]. Another two subspecies, *C. michiganensis*subsp.*californiensis* and *C. michiganensis*subsp.*chilensis* were named to include bacterial isolates from tomato and pepper seeds produced in California and Chile, respectively [7]. Among these newly established subspecies, only *C. michiganensis*subsp.*capsici* with available genome sequence data (Table 1) was used in this study. The other three recently named subspecies were not included in this study.

**Table 1.** Bacterial strains and their genome sequences analysed in this study

Bacterial strains	Strain no	GenBank accession no	Isolated from	Reference
*Clavibacter* sp.	CF 11	JROD01000001	Soil	[22]
*Clavibacter* sp.	LMG 26808	AZQZ01000000	unknown	[12]
*C. michiganensis*subsp.*insidiosus*	LMG 3663^T^	MZMO00000000	Alfalfa	This work
	R1-1	NZ_CP011043	Alfalfa	[23]
*C. m.* subsp. *michiganensis*	LMG 7333^T^	MZMP00000000	Tomato	This work
	NCPPB 382	NC_009480	Tomato	[24]
*C. m.* subsp. *nebraskensis*	NCPPB 2581^T^=LMG 3700^T^	NC_020891	Maize	Gartemann unpublished
	DOAB 397	LAKL01000001	Corn	[25]
	DOAB 395	LSOE01000000	Corn	[21]
*C. m.* subsp. *sepedonicus*	ATCC 33113^T^	NC_010407	Potato	[26]
	CFIA-Cs3N	MZMM00000000	Potato	This work
	CFIA-CsR14	MZMN00000000	Potato	This work
*C. m.* subsp. *tessellarius*	ATCC 33566^T^	MZMQ00000000	Wheat	This work
*C. m.* subsp. *capsici*	PF 008 ^T^	NZ_CP012573	Pepper	[6]
*Leifsonia xyli*subsp.*xyli*	356_LXYL	NZ_JVKI00000000	Sugarcane	[1]
*Leifsonia xyli*subsp*. cynodontis*	DSM 46306	NC_022438	Bermuda Grass	[1]

T, Type strain for the subspecies.

To better define the taxonomic position of the subspecies of *C. michiganensis*, whole-genome sequences of two strains of Cms, six strains of Cmn, two strains of Cmt, and the type strains of Cmm, Cmi, and Cmt were decoded using PacBio single molecule real-time (SMRT) sequencing at Genome Quebec (McGill University and Genome Quebec Innovation Centre, Montreal, Quebec, Canada). The assembled sequences were compared with published sequences of *C. michiganensis*subsp.*michiganensis* and subsp. *insidiosus,* and other clavibacter sequences in GenBank (Table 1). Currently available genome sequences for most type strains of each subspecies of *Clavibacter michiganensis* were included in this study. The genome sequences generated in this study were deposited in Genbank with accession numbers of MZMQ00000000 (Cmt ATCC 33566), MZMM00000000 (Cms CFIA-Cs3N), MZMN00000000 (Cms CFIA-CsR14), MZMO00000000 (Cmi LMG 3663) and MZMP00000000 (Cmm LMG 7333).

Average nucleotide identity (ANI) values of whole genomes represent the degree of identity/similarity between homologous regions shared by two genomes and has emerged as a powerful genome-based criterion for establishing species identity amongst genetically related micro-organisms [8, 9]. The approach evaluates a large number of genes, including both slow and fast evolving genes, in the calculation and thus minimizes the effect of variable evolutionary rates or horizontal gene transfer events [9]. In this study, ANI was calculated using the JSpecies software [10] with the Nucleotide MUMmer algorithm (NUCmer) and default parameter settings. The degree of pairwise genome-based relatedness was calculated as an ANI value following the blast-based ANI calculation method described by Goris *et al*. [11]. ANI was calculated based on comparisons between all strains sequenced in this study and those sequenced previously (Table 1).

The ANI values among the subspecies of *Clavibacter* were generally below the 96 % cutoff value for species delineation suggested by Richter and Rosselló-Móra [10]. ANI values between subspecies were 89.18–95.01 %, whereas ANI values between strains of the same subspecies were >99 % (99.17–99.98 %) (Table 2). Comparative ANI scores of ~90 % for the two strains, CF 11 and LMG 26808, tentatively identified as non-pathogenic isolates of *Clavibacter michiganensis* [12], were well below the 96 % cutoff for species delineation. The taxonomic status of these strains requires further study.

**Table 2.** Average nucleotide identity (ANI; lower diagonal) and digital DNA–DNA hybridization (dDDH; upper diagonal) values among *Clavibacter michiganensis* and related species and subspecies Cut-off values for species delineation are 96.0 and 70.0 % for ANI and dDDH, respectively.

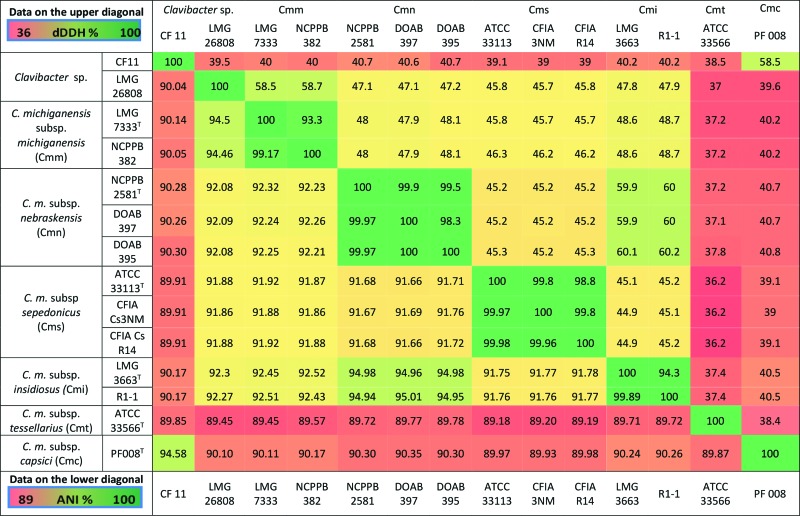

While ANI represents core genome homology, genome–genome distance calculation (GGDC) or digital DNA–DNA hybridization (dDDH) [13, 14] measures the genome-to-genome distances between pairs of entirely or partially sequenced genomes. The digital pairwise estimator for the relatedness of genomes serves as an *in silico* replacement for the wet-lab based DNA–DNA hybridization. In this study dDDH values were calculated using GGDC 2.0 server (http://ggdc.dsmz.de/distcalc2.php) by means of genome-to-genome sequence comparison and pairwise dDDH values were estimated using the GGDC calculator [14]. Consistency with ANI data and dDDH values clearly differentiated the *Clavibacter* subspecies into distinct clades with high degree of congruency with genomospecies allocation (Table 2). The dDDH values between different subspecies were within the range of 37–60 % (Table 2), below the suggested 70 % cut-off for species delineation [14]. Significantly, but not unexpectedly, evaluations between strains of the same subspecies showed dDDH values of more than 93 % (Table 2).

Multi-locus sequence analysis (MLSA) based on concatenated segments of housekeeping genes is used in phylogenetic studies to resolve taxonomic relationships among closely related species [15–17]. MLSA was employed on seven housekeeping genes, *acnA, gapA, lcdA, mdh, mtlD, pgi* and *proA* (Fig. 1). Strains within each of the five *C. michiganensis* subspecies clearly formed five distinct phylogenetic clusters, well-supported by high bootstrap values (Fig. 1). The grouping coincided perfectly with the five apparent genomospecies based on ANI and dDDH values (Table 2). Of the two non-pathogenic strains, LMG 26808 clustered most closely to *C. m.* subsp. *michiganensis* but separate from CF11, which formed a unique cluster. In addition, single gene phylogenies confirmed the distinct clustering of the five subspecies studied (Fig. S1, available in the online version of this article).

**Fig. 1.**
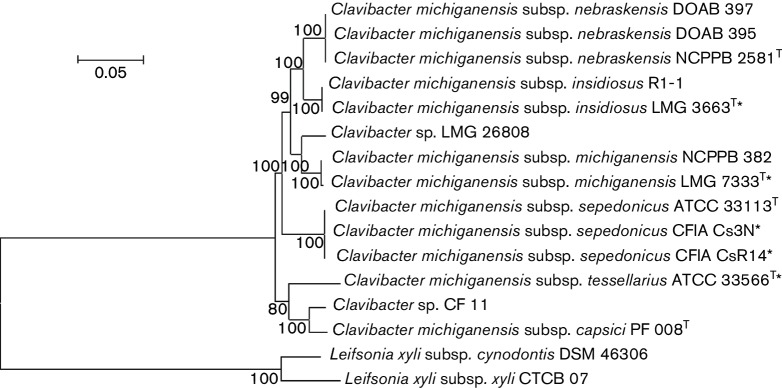
Phylogenetic relationship of *Clavibacter* clades on the basis of multi-locus sequence analysis (MLSA) of concatenated (*acn*A-*gap*A-*icd*A-*mdh-mtl*D-*pgi-pro*A) sequences of the seven housekeeping genes. The evolutionary distances were computed using the Judes–Cantor method with bootstrap value of 100 (>50 are shown). Phylogenetic analysis was conducted in mega6 [27]. *Leifsonia xyli* serves as the out group. *, Current work; T, type strain.

Re-classifying *C. michiganensis* subspecies does not undermine classification based on phenotypic characterization of this group of plant pathogenic bacteria but rather supports their classification as individual species which are easily differentiated by classical bacteriological methods as previously reported [5, 18, 19]. As already noted, each of the *C. michiganensis* subspecies is highly host-specific and in culture can also be readily differentiated by colony pigmentation on many commonly used growth media and substrate utilization (Table 3). Biochemical and physiological test reactions also differentiate each of the *Clavibacter* groups (Table 3).

**Table 3.** Phenotypic characteristics of *Clavibacter michiganensis* subspecies [5, 6, 18, 19]

Characteristic	*C. michiganensis*subsp.*michiganensis*	*C. m.* subsp. *insidiosus*	*C. m.* subsp. *nebraskensis*	*C. m.* subsp. *sepedonicus*	*C. m.* subsp. *tessellarius*	*C. m.* subsp. *capsici*
Major host plant	Tomato	Alfalfa	Maize	Potato	Wheat	Pepper
Colony pigment	Yellow*	Yellow/blue	Orange/yellow	White	Orange	Orange
Colony type	Fluidal	Fluidal	Domed, mucoid	Fluidal	Domed, mucoid	Mucoid
Growth on CNS	+	−	+	−	+	n/a
Growth on TTC	+	+	−	−	+	+
Gelatin liquefaction	+	−	−	−	−†	n/a
Levan production	−	−	+	−	+	+
Acid from sorbitol	−	−	+	+	+	n/a
Acid from mannitol	−	−	−	+	+†	n/a
Utilization of melibiose	+	−	+	−	−	+
Utilization of trehalose	w	+	+	+	+	+
Utilization of fucose	+	−	−	−	−	−
Utilization of acetate	+	−	+	+	−	n/a
Utilization of glycerol	+	+	+	−	+	n/a
Utilization of succinate	+	−	+	+	−†	n/a
Hydrolysis of aesculin	+	+	+	+	+	n/a
Alkaline phosphatase activity	+	−	+	±	+	+
α-Mannosidase activity	−	+	−	−	−	w

CNS, *Corynebacterium nebraskense* semi-selective medium [28]; TTC, 2,3,5 triphenyl tetrazolium chloride medium [29].

*Also various other pigments (e.g. pink, red, orange, white or colourless).

†This work; w, less than 50 % positive results; n/a, not available.

Traditional classification of plant pathogens faces critical challenges in the genome era as sequence data become routinely accessible through next-generation sequencing methods. The growing number of sequenced genomes of plant pathogens provides a rich source of information for new approaches to resolve complex taxonomic questions. In this study, the draft genomes of three type strains of *Clavibacter* species/subspecies, not previously available, were generated and compared with all publicly available GenBank entries so as to accurately define the taxonomic status of the five subspecies within *C. michiganensis*. On the basis of the genome data (ANI and dDDH values) and multi-locus phylogenetic analysis presented in this paper and previously reported phenotypic characteristics, we propose that the bacteria presently classified as *Clavibacter michiganensis* subsp. *capsici* Oh *et al*. 2016, *Clavibacter michiganensis* subsp. *nebraskensis* (Vidaver and Mandel 1974) Davis *et al*. 1984, *Clavibacter michiganensis* subsp. *insidiosus* (McCulloch 1925) Davis *et al*. 1984, *Clavibacter michiganensis* subsp. *sepedonicus* (Spieckermann and Kotthoff 1914) Davis *et al*. 1984, and *Clavibacter michiganensis* subsp. *tessellarius* (Carlson and Vidaver 1982) Davis *et al*. 1984 be reclassified as *Clavibacter capsici* sp. nov., comb. nov.*, Clavibacter nebraskensis* comb. nov., *Clavibacter insidiosus* comb. nov., *Clavibacter sepedonicus* comb. nov., and *Clavibacter tessellarius* sp. nov., comb. nov., respectively. The original type strains of the subspecies become type strains for each of the new species and species descriptions remain the same as for the former descriptions of corresponding subspecies [20].

## Description of *Clavibacter capsici* sp. nov., comb. nov.

*Clavibacter capsici* (cap′si.ci. N.L. neut. gen. n. *capsici*, referring to *Capsicum*, the genus name of pepper).

Basonym: *Clavibacter michiganensis*subsp.*capsici* Oh *et al.* 2016.

The species description is unchanged from its description as *Clavibacter michiganensis*subsp.*capsici* given by Oh *et al.* [6].

The type strain is PF008^T^ (=KACC 18448^T^=LMG 29047^T^). The type strain was originally isolated from pepper showing bacterial canker disease in Anyang, Republic of Korea.

## Description of *Clavibacter insidiosus* comb. nov.

*Clavibacter insidiosus* (in.si.di.o′sus. L. masc. adj. *insidiosus*, deceitful, insidious).

Basonym: *Corynebacterium insidiosum* (McCulloch 1925) Jensen 1934, *Corynebacterium michiganense*subsp.*insidiosum* (McCulloch 1925) Carlson and Vidaver 1982, *Clavibacter michiganensis*subsp*insidiosus* (McCulloch 1925) Davis *et al*. 1984.

Gram-stain-positive, non-spore forming, aerobic bacterium without flagella. Produces yellowish colonies with blue granules on common laboratory growth media. Grows on TTC but not CNS medium. It does not liquefy gelatin nor produces levan. It does not produce acid from either sorbitol or mannitol. It utilizes glycerol but not acetate or succinate; it hydrolyses aesculin, and has α-mannosidase activity but no alkaline phosphatase activity. It causes bacterial wilt disease of alfalfa (lucerne) (*Medicago sativa* L.). DNA G+C content of the type strain is 72.7 %. The type strain is LMG 3663^T^ (=ATCC 10253^T^=NCPPB1109^T^).

## Description of *Clavibacter nebraskensis* comb. nov.

*Clavibacter nebraskensis* (ne.bras.ken′sis. N.L. masc. adj. *nebraskensis*, pertaining to the state of Nebraska, USA).

Basonym: *Corynebacterium nebraskense* Vidaver and Mandel 1974, *Corynebacterium michiganense*subsp.*nebraskense* (Vidaver and Mandel 1974) Carlson and Vidaver 1982, *Clavibacter michiganensis*subsp.*nebraskensis* (Vidaver and Mandel 1974) Davis *et al*. 1984.

Gram-stain-positive, non-spore forming, aerobic bacterium without flagella. Produces yellow to orange colonies on common laboratory growth media. It grows on CNS but does not grow on TTC medium. It does not liquefy gelatin but it does produce levan. It produces acid from sorbitol but it does not produce acid from mannitol. It utilizes acetate, glycerol and succinate. It hydrolyses aesculin, it has alkaline phosphatase activity, but it does not have α-mannosidase activity. It causes leaf freckles and a wilt disease of maize (*Zea mays* L.) DNA G+C content of the type strain is 73.0 %. The type strain is NCPPB 2581^T^ (=ATCC 27794^T^=LMG 3700^T^).

## Description of *Clavibacter sepedonicus* comb. nov.

*Clavibacter sepedonicus* (se.pe.do′ni.cus. Gr. n. *sepedon* rottenness, decay; N.L. masc. adj. *sepedonicus*, leading to decay).

Basonym: *Corynebacterium sepedonicum* (Spieckermann and Kotthoff 1914) Skaptason and Burkholder 1942, *Corynebacterium michiganense*subsp.*sepedonicum* (Spieckermann and Kotthoff 1914) Carlson and Vidaver 1982, *Clavibacter michiganensis*subsp.*sepedonicus* (Spieckermann and Kotthoff 1914) Davis *et al*. 1984.

Gram-stain-positive, non-spore forming, aerobic bacterium without flagella. Produces white mucoid colonies at an optimum growth temperature of 20–23 °C. It does not grow on CNS or TTC media. It does not liquefy gelatin nor produces levan. It differs from the other *Clavibacter* species in producing acid from both sorbitol and mannitol. It utilizes acetate and succinate but not glycerol; it hydrolyses aesculin; alkaline phosphatase activity is weak, and α-mannosidase activity is lacking. It causes bacterial ring rot disease of potato (*Solanum tuberosum* L). DNA G+C content of the type strain is 72.4 %. The type strain is ATCC 33113^T^ (=LMG 2889^T^=NCPPB 2137^T^).

## Description of *Clavibacter tessellarius* sp. nov. comb. nov.

*Clavibacter tessellarius* (tes.sel.la′ri.us. L. masc. n. *tessellarius* a mosaic stone maker).

Basonym: *Clavibactermichiganense*subsp.*tessellarius, Clavibacter michiganensis*subsp.*tessellarius* (Carlson and Vidaver 1982) Davis *et al*. 1984.

The species description is unchanged from its description as *Clavibacter michiganensis*subsp.*tessellarius* given by Carlson and Vidaver, 1982 [20].

The type strain is ATCC 33566^T^ (=NCPPB 3664^T^=LMG 7294^T^).

This new taxonomy not only resolves the long-standing problem of having only a single species within the well-established genus, *Clavibacter*, but it also provides a practical solution for plant pathologists and policy makers dealing with quarantine and regulated plant pathogens. *C. michiganensis, C. sepedonicus* and *C. insidiosus* are quarantine or regulated pathogens of important agricultural crops in many countries, while *C. capsici* is a newly described plant pathogen for which the range of distribution and risk to agriculture need to be assessed. The revised classification, and accordingly a simpler nomenclature, uncomplicates regulatory documents and more accurately reflects biological reality.

While this manuscript was under review, one of the co-authors [21] of this manuscript carried out an independent investigation titled ‘Comparative genomics of *Clavibacter michiganensis* subspecies, pathogens of important agricultural crops’. It is quoted here ‘the study also assessed the taxonomic position of the subspecies based on 16S rRNA and genome-based DNA homology and concludes that there is ample evidence to elevate some of the subspecies to species-level’.

## Supplementary Data

50 Supplementary File 1
