# Supplementary material for: Re-classification of Clavibacter michiganensis subspecies on the basis of whole-genome and multi-locus sequence analyses
Source: Int J Syst Evol Microbiol. 2017 Nov 21;68(1):234–40. doi: 10.1099/ijsem.0.002492 (PMC5882085; doi:10.1099/ijsem.0.002492)
Supplement: Supplementary File 1 [file ijsem-68-234-s001.pdf]

Supplementary materials

Figure S1. Single gene phylogenetic trees: *acnA* (a), *gapA* (b), *icdA* (c), *mdh* (d), *md1D* (e), *pgi* (f), and *proA* (g) showing relationship of the *Clavibacter* clades of the seven house-keeping gene sequences. *Leifsonia xyli* serves as the out group. *: Current work. T: Type strain.

(a), *acnA*

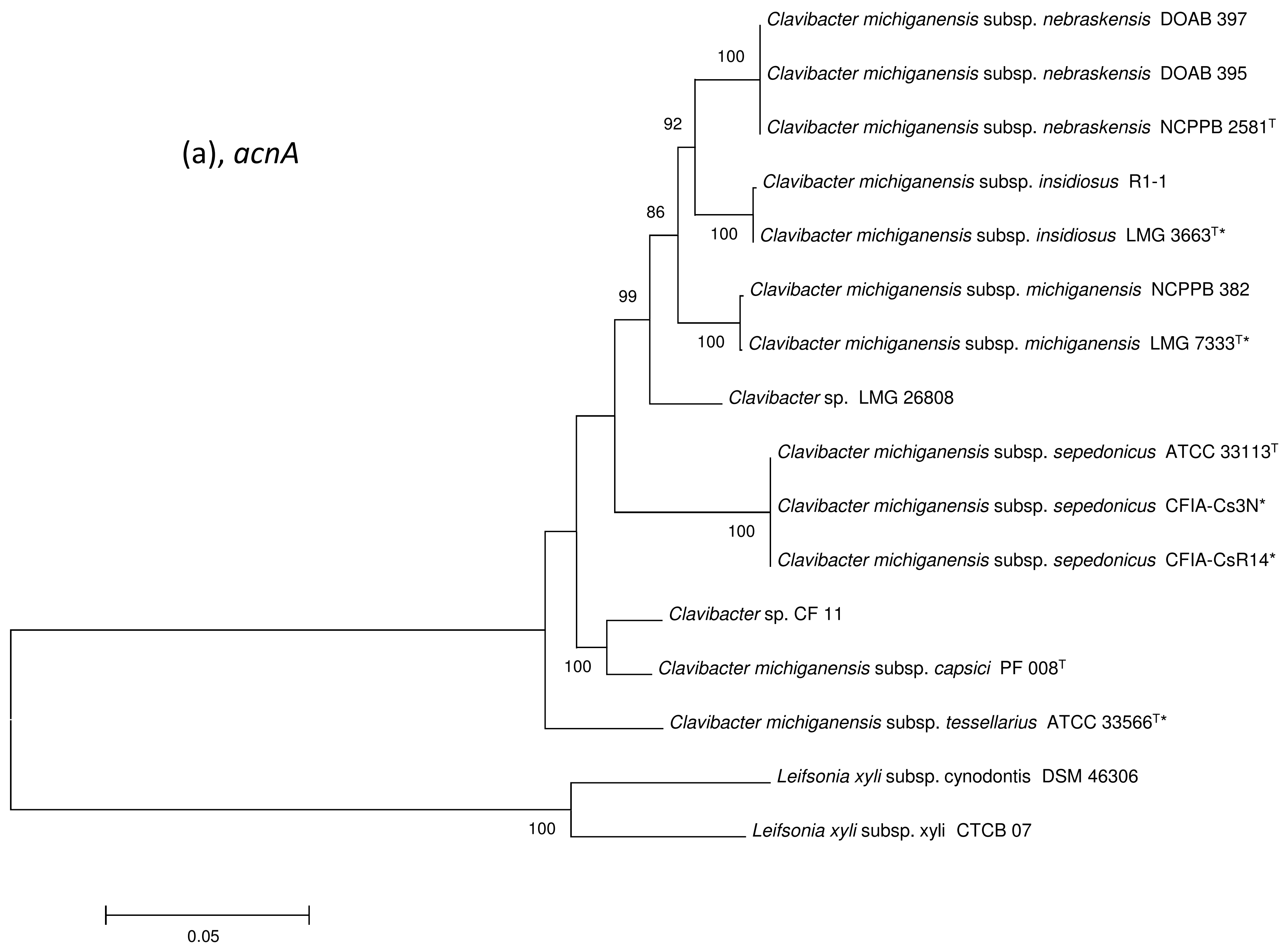
Clavibacter michiganensis subsp. nebraskensis DOAB 397
Clavibacter michiganensis subsp. nebraskensis DOAB 395
Clavibacter michiganensis subsp. nebraskensis NCPPB 2581T
Clavibacter michiganensis subsp. insidiosus R1-1
Clavibacter michiganensis subsp. insidiosus LMG 3663T*
Clavibacter michiganensis subsp. michiganensis NCPPB 382
Clavibacter michiganensis subsp. michiganensis LMG 7333T*
Clavibacter sp. LMG 26808
Clavibacter michiganensis subsp. sepedonicus ATCC 33113T
Clavibacter michiganensis subsp. sepedonicus CFIA-Cs3N*
Clavibacter michiganensis subsp. sepedonicus CFIA-CsR14*
Clavibacter sp. CF 11
Clavibacter michiganensis subsp. capsici PF 008T
Clavibacter michiganensis subsp. tessellarius ATCC 33566T*
Leifsonia xyli subsp. cynodontis DSM 46306
Leifsonia xyli subsp. xyli CTCB 07
100
92
100
86
100
99
100
100
100
100
0.05

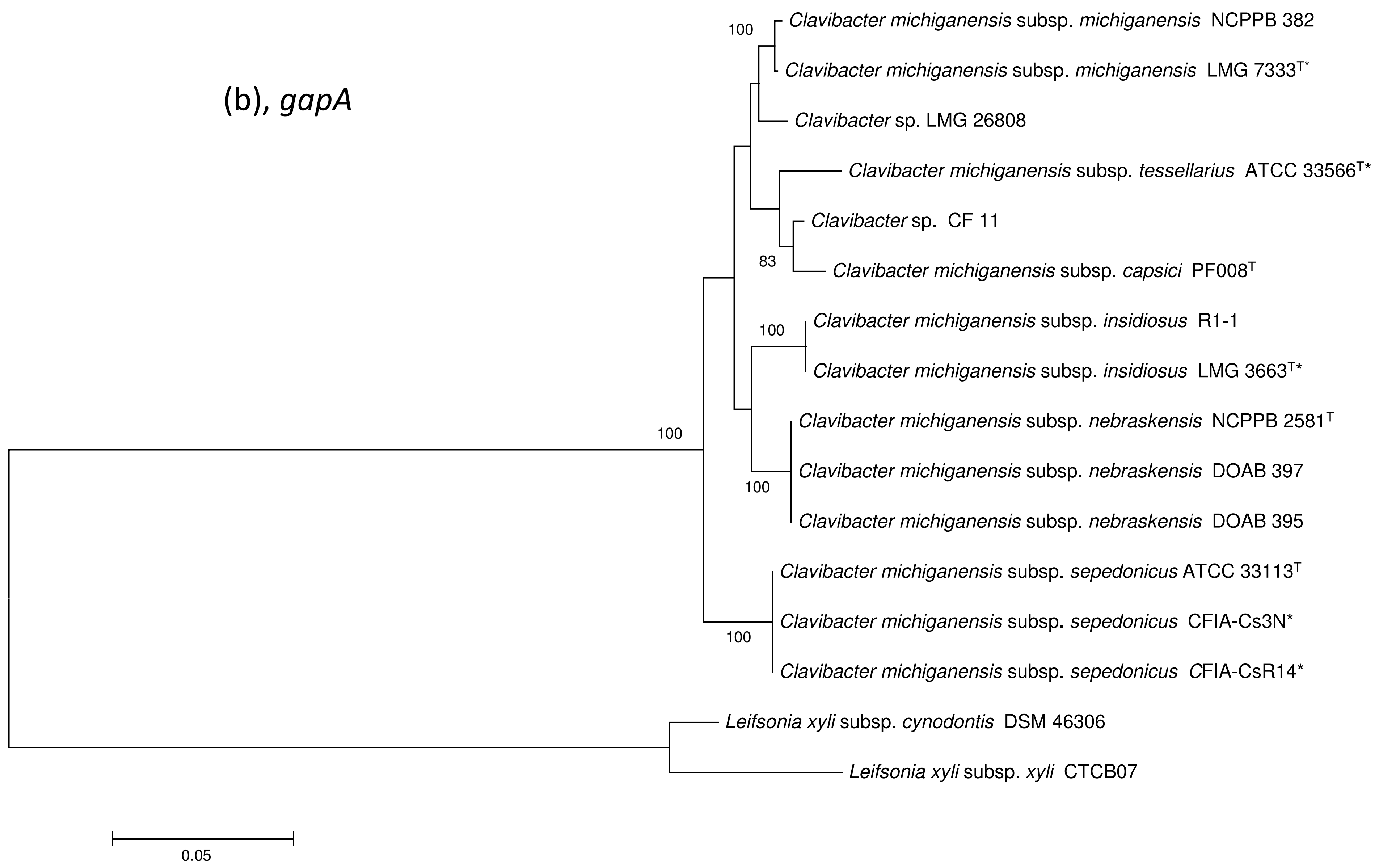
(b), gapA
Clavibacter michiganensis subsp. michiganensis NCPPB 382
Clavibacter michiganensis subsp. michiganensis LMG 7333T*
Clavibacter sp. LMG 26808
Clavibacter michiganensis subsp. tessellarius ATCC 33566T*
Clavibacter sp. CF 11
Clavibacter michiganensis subsp. capsici PF008T
Clavibacter michiganensis subsp. insidiosus R1-1
Clavibacter michiganensis subsp. insidiosus LMG 3663T*
Clavibacter michiganensis subsp. nebraskensis NCPPB 2581T
Clavibacter michiganensis subsp. nebraskensis DOAB 397
Clavibacter michiganensis subsp. nebraskensis DOAB 395
Clavibacter michiganensis subsp. sepedonicus ATCC 33113T
Clavibacter michiganensis subsp. sepedonicus CFIA-Cs3N*
Clavibacter michiganensis subsp. sepedonicus CFIA-CsR14*
Leifsonia xyli subsp. cynodontis DSM 46306
Leifsonia xyli subsp. xyli CTCB07
100
83
100
100
100
100
0.05

(c), *icdA*

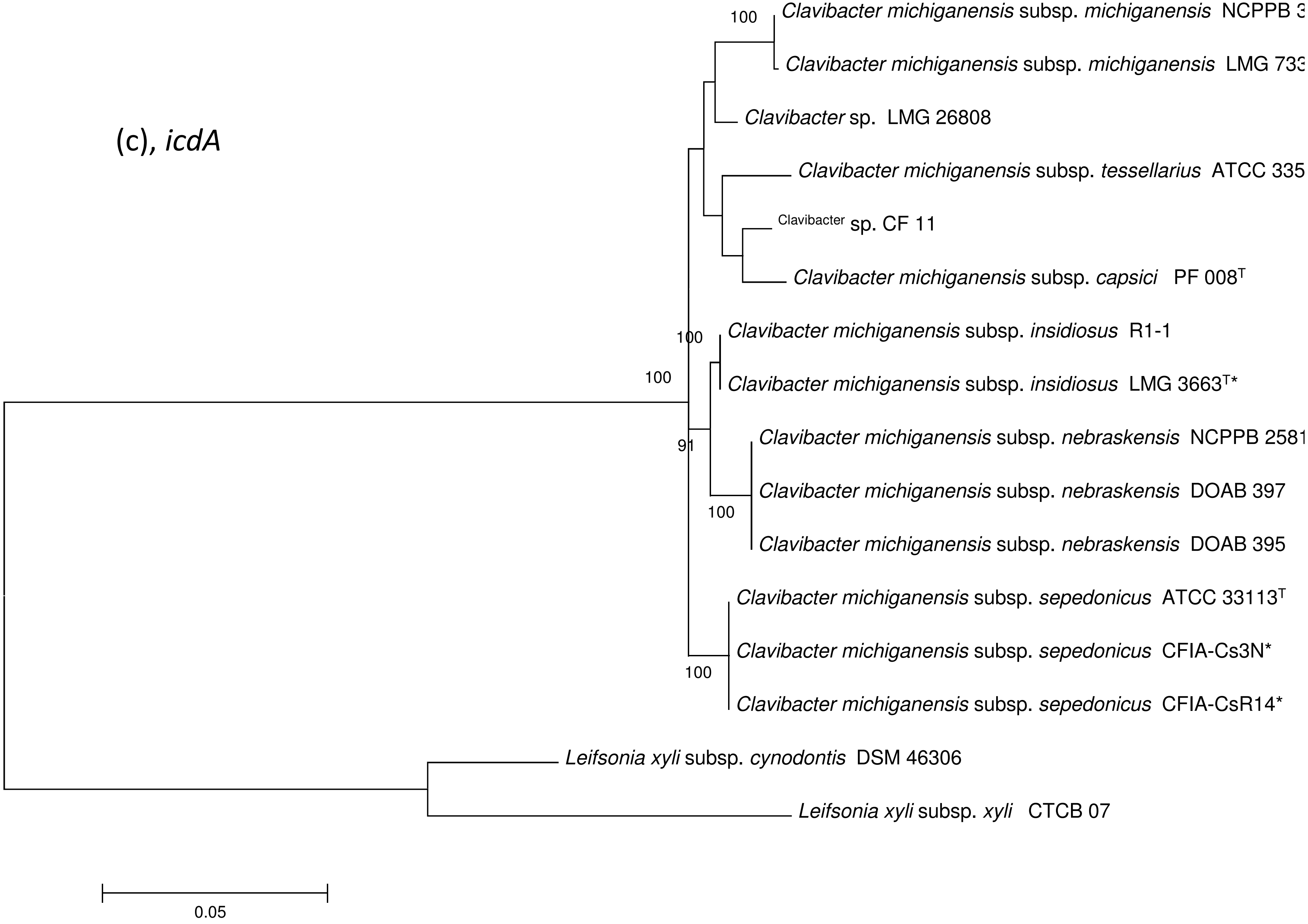

Clavibacter michiganensis subsp. michiganensis NCPPB 3
Clavibacter michiganensis subsp. michiganensis LMG 733
Clavibacter sp. LMG 26808
Clavibacter michiganensis subsp. tessellarius ATCC 335
Clavibacter sp. CF 11
Clavibacter michiganensis subsp. capsici PF 008T
Clavibacter michiganensis subsp. insidiosus R1-1
Clavibacter michiganensis subsp. insidiosus LMG 3663T*
Clavibacter michiganensis subsp. nebraskensis NCPPB 2581
Clavibacter michiganensis subsp. nebraskensis DOAB 397
Clavibacter michiganensis subsp. nebraskensis DOAB 395
Clavibacter michiganensis subsp. sepedonicus ATCC 33113T
Clavibacter michiganensis subsp. sepedonicus CFIA-Cs3N*
Clavibacter michiganensis subsp. sepedonicus CFIA-CsR14*
Leifsonia xyli subsp. cynodontis DSM 46306
Leifsonia xyli subsp. xyli CTCB 07
100
100
100
91
100
100
0.05

(d), *mdh*

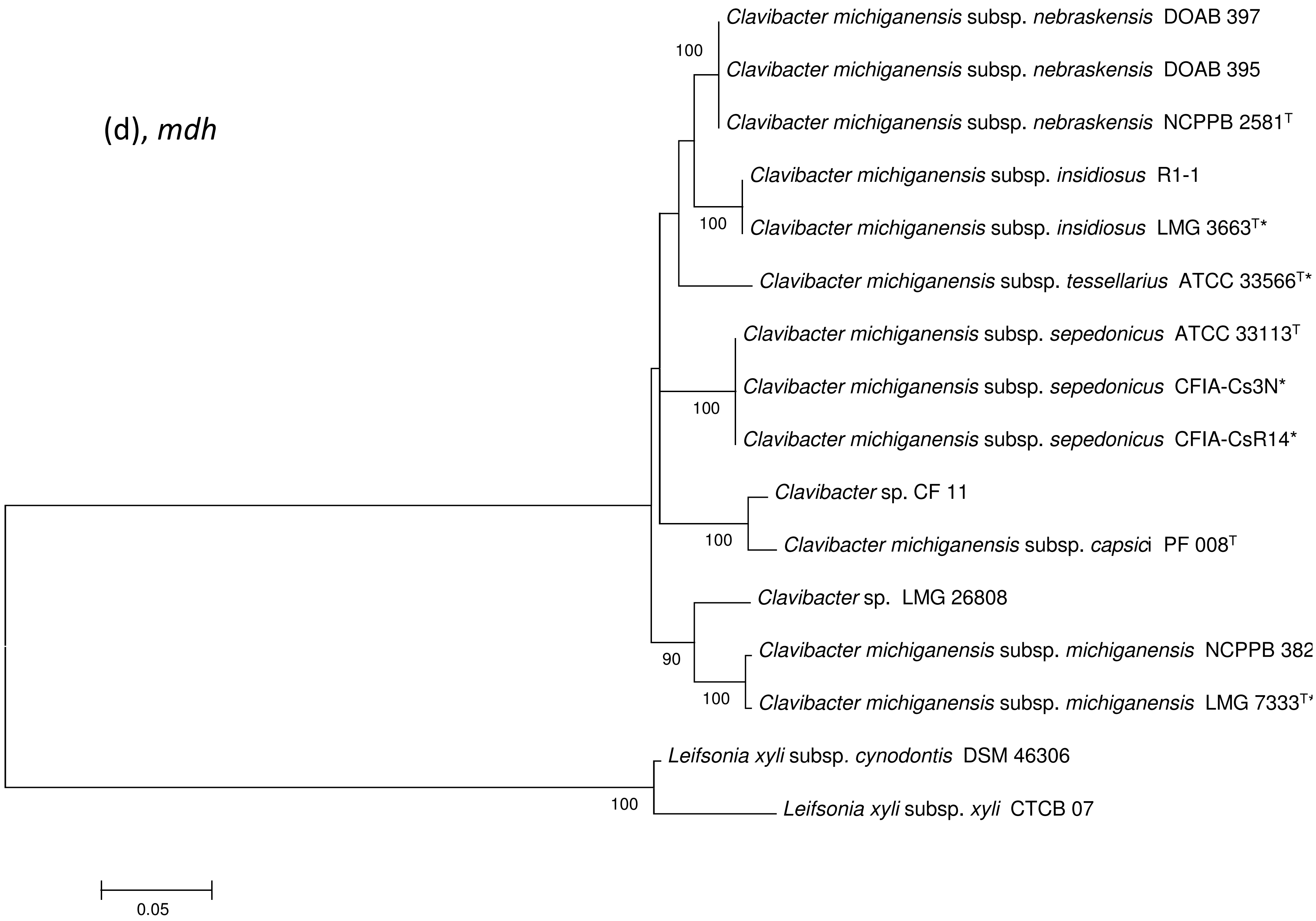

100
Clavibacter michiganensis subsp. nebraskensis DOAB 397
Clavibacter michiganensis subsp. nebraskensis DOAB 395
Clavibacter michiganensis subsp. nebraskensis NCPPB 2581T
Clavibacter michiganensis subsp. insidiosus R1-1
100
Clavibacter michiganensis subsp. insidiosus LMG 3663T*
Clavibacter michiganensis subsp. tessellarius ATCC 33566T*
Clavibacter michiganensis subsp. sepedonicus ATCC 33113T
Clavibacter michiganensis subsp. sepedonicus CFIA-Cs3N*
100
Clavibacter michiganensis subsp. sepedonicus CFIA-CsR14*
Clavibacter sp. CF 11
100
Clavibacter michiganensis subsp. capsici PF 008T
Clavibacter sp. LMG 26808
Clavibacter michiganensis subsp. michiganensis NCPPB 382
90
100
Clavibacter michiganensis subsp. michiganensis LMG 7333T*
Leifsonia xyli subsp. cynodontis DSM 46306
100
Leifsonia xyli subsp. xyli CTCB 07
0.05

(e), *mt1D*

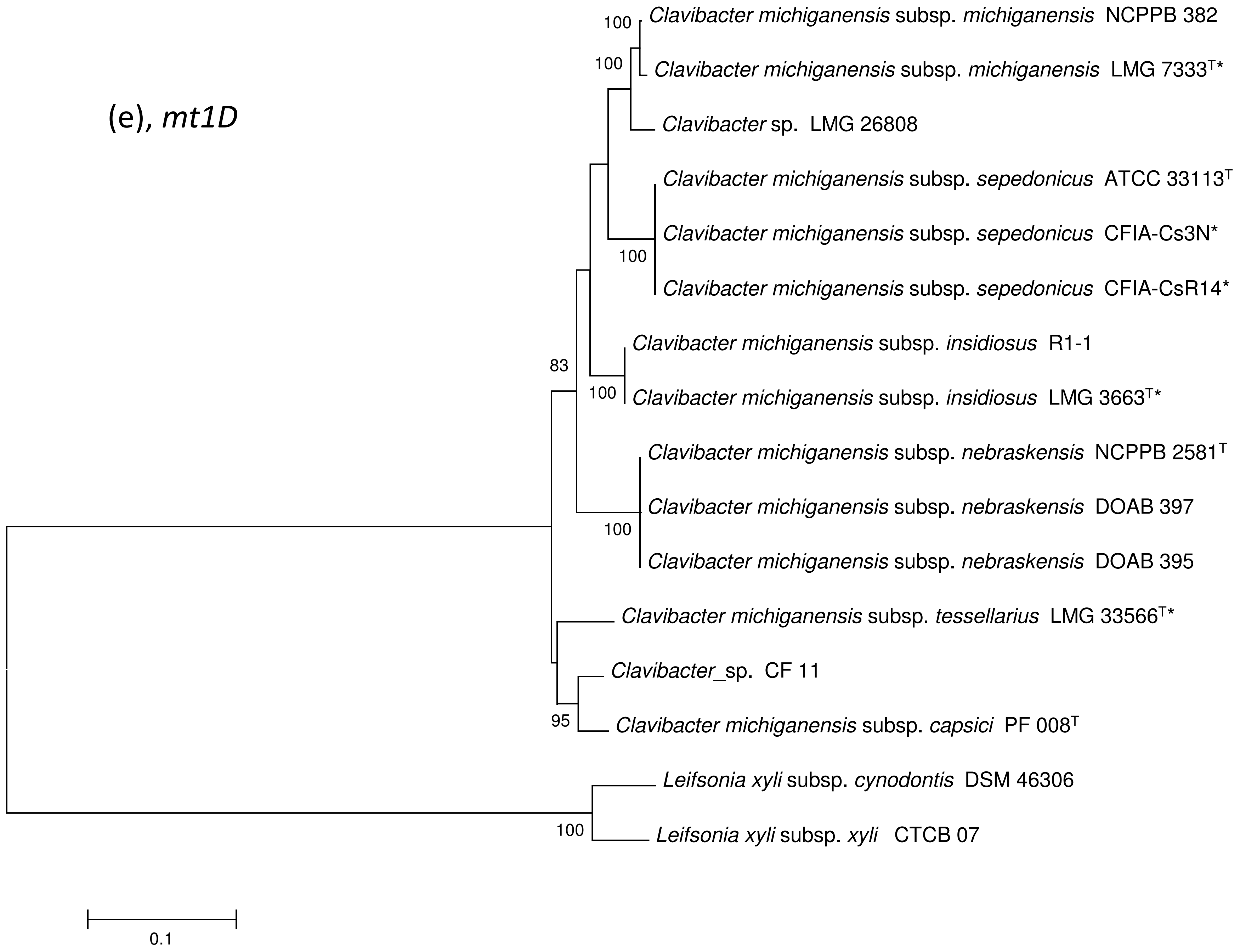

100
100
Clavibacter michiganensis subsp. michiganensis NCPPB 382
Clavibacter michiganensis subsp. michiganensis LMG 7333T*
Clavibacter sp. LMG 26808
Clavibacter michiganensis subsp. sepedonicus ATCC 33113T
Clavibacter michiganensis subsp. sepedonicus CFIA-Cs3N*
Clavibacter michiganensis subsp. sepedonicus CFIA-CsR14*
100
83
Clavibacter michiganensis subsp. insidiosus R1-1
Clavibacter michiganensis subsp. insidiosus LMG 3663T*
100
Clavibacter michiganensis subsp. nebraskensis NCPPB 2581T
Clavibacter michiganensis subsp. nebraskensis DOAB 397
Clavibacter michiganensis subsp. nebraskensis DOAB 395
100
Clavibacter michiganensis subsp. tessellarius LMG 33566T*
Clavibacter_sp. CF 11
Clavibacter michiganensis subsp. capsici PF 008T
95
Leifsonia xyli subsp. cynodontis DSM 46306
Leifsonia xyli subsp. xyli CTCB 07
100
0.1

(f), *pgi*

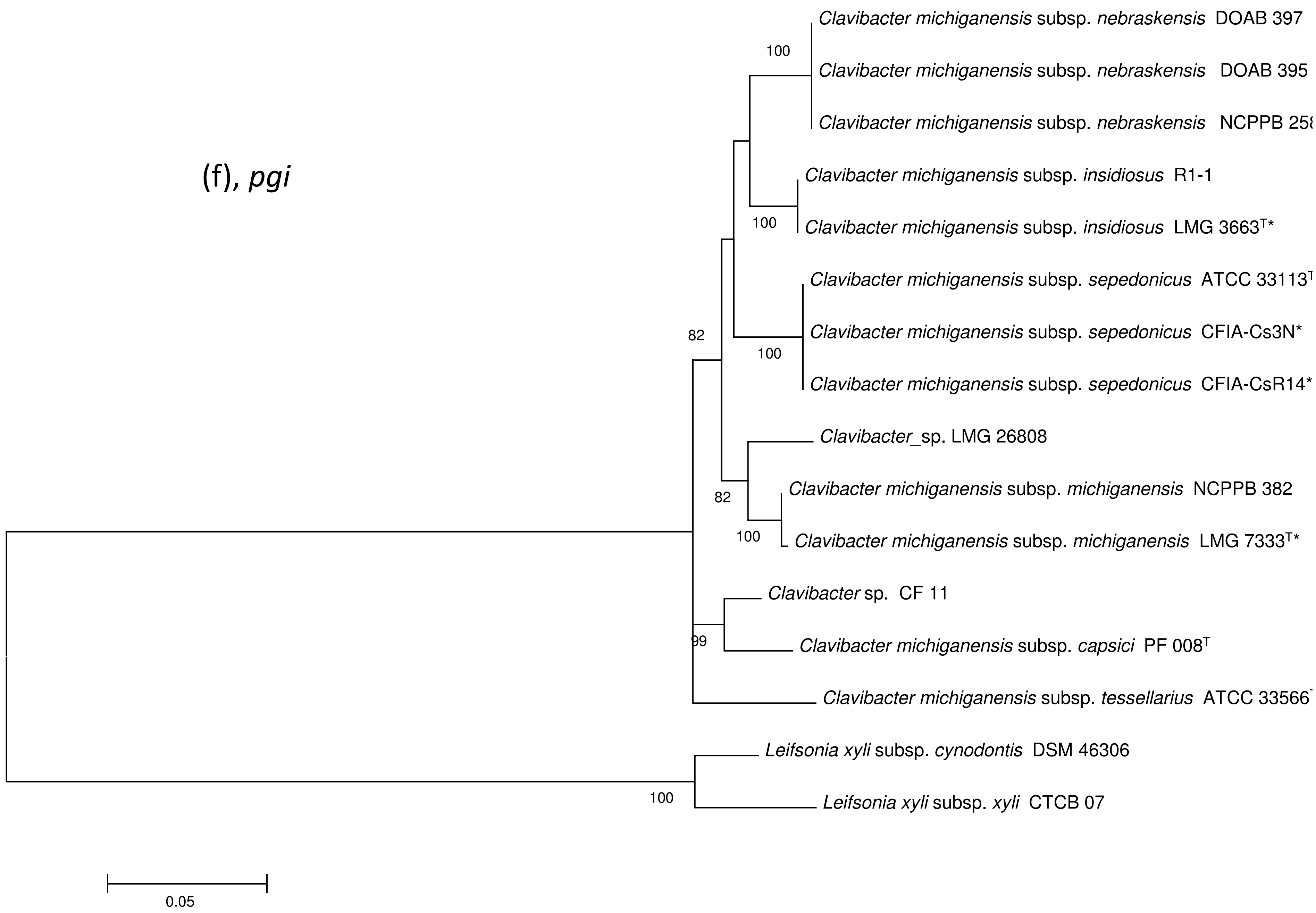
Clavibacter michiganensis subsp. nebraskensis DOAB 397
Clavibacter michiganensis subsp. nebraskensis DOAB 395
Clavibacter michiganensis subsp. nebraskensis NCPPB 25
Clavibacter michiganensis subsp. insidiosus R1-1
Clavibacter michiganensis subsp. insidiosus LMG 3663T*
Clavibacter michiganensis subsp. sepedonicus ATCC 33113
Clavibacter michiganensis subsp. sepedonicus CFIA-Cs3N*
Clavibacter michiganensis subsp. sepedonicus CFIA-CsR14*
Clavibacter_sp. LMG 26808
Clavibacter michiganensis subsp. michiganensis NCPPB 382
Clavibacter michiganensis subsp. michiganensis LMG 7333T*
Clavibacter sp. CF 11
Clavibacter michiganensis subsp. capsici PF 008T
Clavibacter michiganensis subsp. tessellarius ATCC 33566
Leifsonia xyli subsp. cynodontis DSM 46306
Leifsonia xyli subsp. xyli CTCB 07
100
100
100
82
82
100
99
100
0.05

(g), *proA*

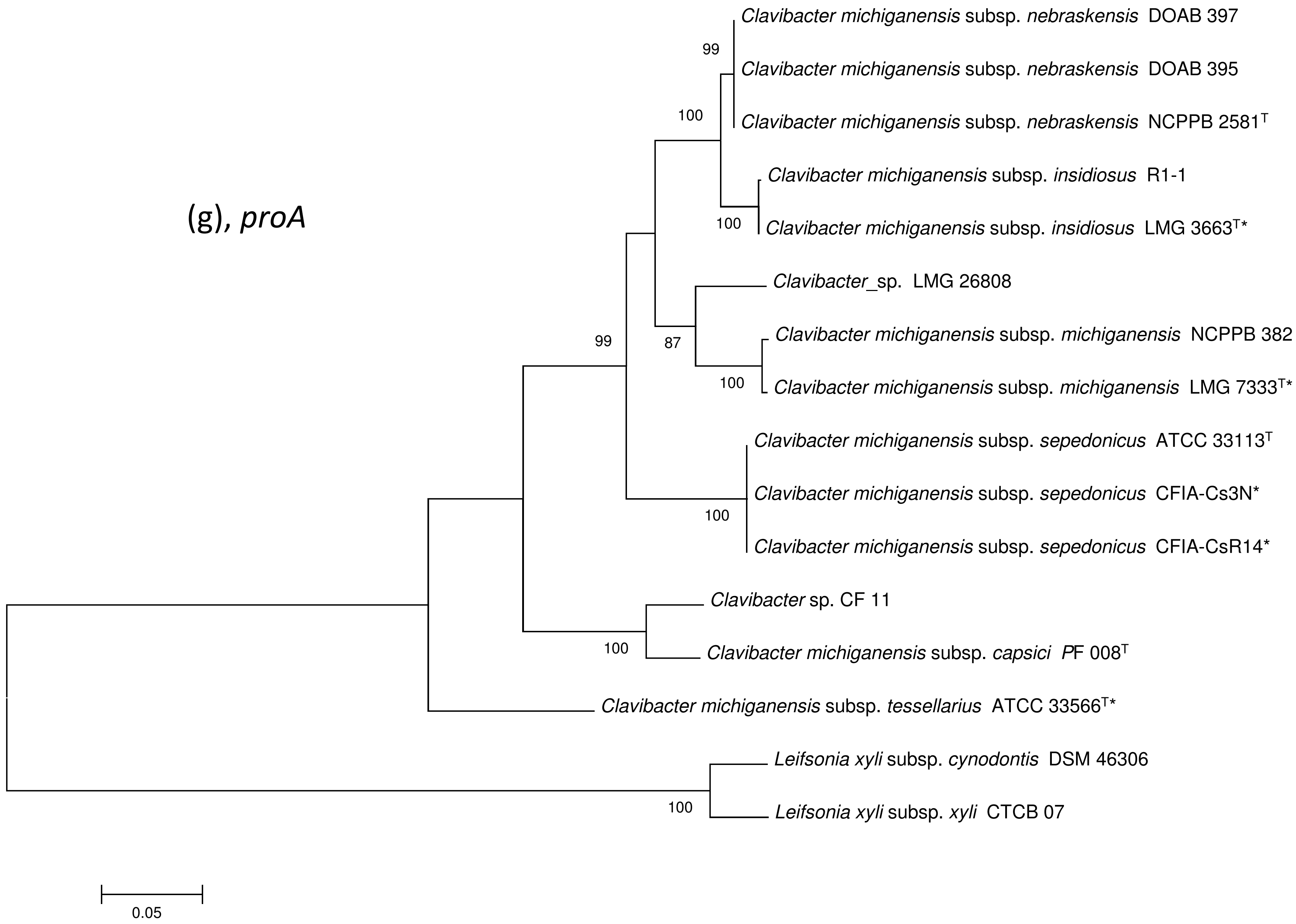
Clavibacter michiganensis subsp. nebraskensis DOAB 397
Clavibacter michiganensis subsp. nebraskensis DOAB 395
Clavibacter michiganensis subsp. nebraskensis NCPPB 2581T
Clavibacter michiganensis subsp. insidiosus R1-1
Clavibacter michiganensis subsp. insidiosus LMG 3663T*
Clavibacter_sp. LMG 26808
Clavibacter michiganensis subsp. michiganensis NCPPB 382
Clavibacter michiganensis subsp. michiganensis LMG 7333T*
Clavibacter michiganensis subsp. sepedonicus ATCC 33113T
Clavibacter michiganensis subsp. sepedonicus CFIA-Cs3N*
Clavibacter michiganensis subsp. sepedonicus CFIA-CsR14*
Clavibacter sp. CF 11
Clavibacter michiganensis subsp. capsici PF 008T
Clavibacter michiganensis subsp. tessellarius ATCC 33566T*
Leifsonia xyli subsp. cynodontis DSM 46306
Leifsonia xyli subsp. xyli CTCB 07
99
100
100
99
87
100
100
100
100
0.05
